# The relationship between Type D personality with atherosclerotic plaque and cardiovascular events: The mediation effect of inflammation and kynurenine/tryptophan metabolism

**DOI:** 10.3389/fcvm.2022.986712

**Published:** 2022-10-17

**Authors:** Yini Wang, Guojie Liu, Zhenjuan Zhao, Ling Li, Shi Yin, Xiao Sun, Bo Yu, Xueqin Gao, Ping Lin, Yanjie Yang

**Affiliations:** ^1^Psychology Department of the Public Health Institute of Harbin Medical University, Harbin, China; ^2^Department of Cardiology, The Second Affiliated Hospital of Harbin Medical University, Harbin, China; ^3^Department of Neurosurgery, The Second Affiliated Hospital of Harbin Medical University, Harbin, China; ^4^Department of Nursing, The Second Affiliated Hospital of Harbin Medical University, Harbin, China

**Keywords:** Type D personality, tryptophan, kynurenine, inflammation, plaque vulnerability, major adverse cardiac events, coronary artery disease

## Abstract

**Purpose:**

Cardiovascular events and coronary plaque vulnerability are linked to Type D personality. However, the fundamental mechanism has not been clarified. Our study determined to illustrate whether inflammatory status in plasma, in combination with kynurenine pathway activity in Type D individuals, is associated with plaque vulnerability and cardiovascular events in patients with coronary artery disease (CAD).

**Materials and methods:**

The Type D personality of 177 CAD patients were evaluated. Plasma biomarkers of inflammation (TNF-α, IL-6, and hs-CRP) were measured and pooled into standardized sumscores. Tryptophan and kynurenine metabolites were measured, and the kynurenine/tryptophan ratio (KTR) was calculated. Plaque vulnerability was measured *in vivo* by optical coherence tomography. All patients had a follow up of 2 years in which cardiovascular adverse events were recorded.

**Results:**

Type D individuals exhibited elevated TNF-α (*p* = 0.007), IL-6 (*p* = 0.049), inflammation sumscores (*p* = 0.002), kynurenine (*p* = 0.008), and KTR (*p* = 0.005) than non-Type D group. The serial-multiple mediation showed that the Type D personality with a direct, favorable impact on plaque vulnerability, including thin cap fibroatheroma (TCFA) (point estimate = 0.81; 95% *CI* = 0.09–1.53), macrophages (point estimate = 0.79; 95% *CI* = 0.05–1.51), and major adverse cardiac events (MACE) (point estimate = 0.88, 95% *CI* = 0.08–1.70). In addition, the standardized inflammation sumscores and KTR were mediators of the Type D personality associations with TCFA, macrophages and MACE.

**Conclusion:**

These results demonstrated that the connection between Type D personality and poor cardiovascular outcomes in CAD patients can be mediated by pro-inflammatory biomarkers and KTR.

## Introduction

Type D (distressed) personality can be distinguished from the interaction of two features: the inclination to express negative feelings and the inhibition of self-expression (1), which may be a role factor for the development and prognosis of coronary artery disease (CAD) (2). Type D personality has been shown to be an independent influence factor of major adverse cardiac events (MACE) in CAD patients (3). In optical coherence tomography (OCT) studies, Type D individuals was shown to have significantly more coronary vulnerable plaques (4, 5). From a coronary imaging perspective, these studies confirmed the effect of Type D on cardiac disease. However, the plausible biological mechanisms between Type D personality, plaque vulnerability, and MACE has not been elucidated.

**FIGURE 1:**
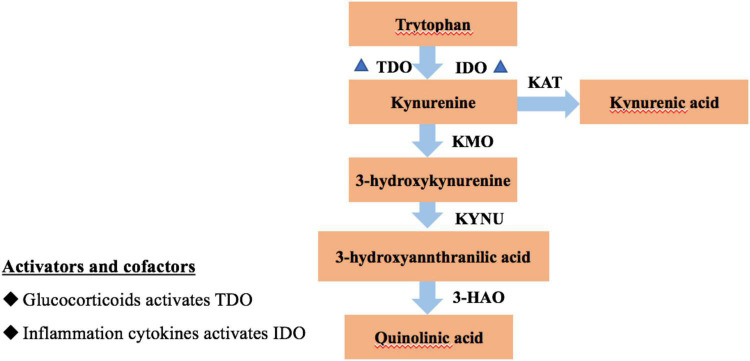
Tryptophan metabolism along the kynurenine pathway.

Evidence suggests that Type D individuals activate the hypothalamic-pituitary adrenal axis and produce high concentrations of the stress hormone, cortisol when they are in response to stress (6). Cortisol controls stress responses and modulates the production of pro-inflammatory cytokines, which is an important link between Type D personality and cardiovascular risk (2). It has been established that stress-related stimulation to the hypothalamic-pituitary-adrenal axis leads to the enzyme tryptophan 2,3-dioxygenase (TDO), the enzyme indoleamine-2,3-dioxygenase (IDO) that triggers the kynurenine pathway, is expressed more readily in response to inflammation, thereby, elevating the production of kynurenine from tryptophan (Figure 1) (7). However, it is not clear if the kynurenine metabolite is a biochemical signature of Type D personality.

Cardiovascular disease progression has been linked to the kynurenine pathway of tryptophan breakdown (8). Eva Ringdal Pedersen et al. found that after 55 months of clinical follow-up, individuals with stable CAD who had elevated kynurenine/tryptophan ratio (KTR) levels had a higher risk of MACE and mortality (9). The IDO expressed in coronary atherosclerotic plaques can increase KTR and enhance tissue factor expression, resulting in thrombus formation (10). It has been proposed that a biomarker of inflammatory states that may affect CAD is KTR. However, it is lack of transformation and application in clinical practice, and it is not clear whether the inflammation activated kynurenine pathway of tryptophan metabolism is the underlying mechanism for development of CAD in Type D personality individuals.

In this study, we aimed at elucidating on the connections between Type D personality, inflammatory variables, tryptophan, and kynurenine metabolites in CAD patients. On the basis of OCT measurement and 2-year follow-up, we explicitly set out to test the idea that the serial multiple mediation effect of inflammation and kynurenine/tryptophan metabolism would cause Type D personality to indirectly affect plaque vulnerability and MACE.

## Materials and methods

### Study participants

Between September 2018 and April 2019, 195 consecutive patients with CAD underwent coronary angiography at the Second Affiliated Hospital of Harbin Medical University were enrolled in this study. We selected individuals in this cohort who were between the ages of 18 and 75. The exclusion criteria were: patients with a history of acute infection, malignancy, and thyroid dysfunction (*n* = 7); patients who had been prescribed with immunosuppressive or corticosteroid drugs within the past 3 months (*n* = 2), positive psychiatric history (*n* = 0), inability to provide informed consent (*n* = 4), failure to complete questionnaires or follow-up (*n* = 5), finally 177 patients were enrolled in the study.

Patients who satisfied the requirements for the study were requested to fill out the questionnaires the following day, after having a selective coronary arteriography assessment and their medical state having stabilized, regardless of whether the patient underwent PCI, and all of them have known the results of angiography. Patient’s clinical data was collected via the health recording systems. The early morning fasting venous blood was collected before coronary angiography. Using hospital records and telephone interviews at 1, 3, 6, 12, and 24 months following discharge, information on the recurrence of angina pectoris, cardiac death, revascularization, and recurrent non-fatal myocardial infarction (MI) was gathered during the follow-up. The Second Affiliated Hospital Ethics Committee of Harbin Medical University provided its ethical approval. Each participant signed an informed consent form in writing.

### Assessment of Type D personality

The 14-item Chinese version of the Type D Scale (DS14) was used to evaluate Type D Personality. The DS14 has two 7-item subscales: social inhibition and negative affectivity (NA) (SI). A Likert scale with five possible outcomes—0 for false, 4 for true—was employed. Each subscale’s cutoff value of ≥10 denotes a Type D personality. NA and SI subscales have Cronbach’s alphas of 0.89 and 0.88, respectively (11).

### Biological measures

#### Measurement of tryptophan and kynurenine

After drawing blood samples into EDTA tubes, they were immediately centrifuged for 15 min at 2,000 G and 4°C. Aliquots were kept at –80°C in preparation for further analysis. Magigene^1^ measured plasma levels of tryptophan and kynurenine using high-performance liquid chromatography coupled with mass spectrometry. KTR was calculated as $\left[\text{KYN}\,\left(\frac{n\,m\,o\,l}{L}\right)÷\text{TRP}\,\left(\frac{u\,m\,o\,l}{L}\right)\right]\times 100$ (12).

#### Inflammatory markers

Tumor necrosis factor-a (TNF-α), interleukin (IL)-6, and hs-C reactive protein (hs-CRP) were among the inflammatory markers measured. Enzyme-linked immunosorbent assays were used to measure the concentrations of hs-CRP (analytical sensitivity: 0.05 mg/l), TNF-α (analytical sensitivity: 4.69 pg/ml), and IL-6 (analytical sensitivity: 4.69 pg/ml) at the major laboratory of myocardial ischemia.^2^ Overall inflammation standardized sumscores were computed as follows for each individual biomarker in order to maximize statistical efficiency and minimize the impact of biological variability on each measure: [individual valuepopulation mean]population standard deviation. TNF-α, IL-6, and hs-CRP were then added to the individual biomarker z-scores to provide an overall standardized inflammatory sumscores (13).

#### Optical coherence tomography image acquisition and analysis

With the help of the C7-XR/ILUMIEN OCT system, OCT imaging was carried out. Two seasoned researchers who were blinded to the patient information independently examined OCT photographs that had been digitally stored in a database. OCT was utilized in this investigation to examine *in vivo* plaque vulnerability features. As previously mentioned, measurements were carried out (14). In a nutshell, lipid rich plaque was defined as a lipid plaque with a lipid arc of less than 90 degrees around the vessel wall. To determine a mean value, the minimal fibrous cap thickness was measured in triplicate at the thinnest spot. A lipid-rich plaque with a fibrous cap thickness of less than 65 μm is known as a thin cap fibroatheroma (TCFA). Macrophage infiltration was recognized as areas of high signal intensity and diverse backward shadows. Rupture was also noted when the plaque’s fibrous top broke down and a hollow formed (13). A mass that protrudes irregularly into the vessel lumen and has a diameter of more than 250 m is referred to as a thrombus.

#### Cardiovascular outcomes

MACE, which included recurrent non-fatal myocardial infarction, revascularization, and cardiac mortality at 2-year follow-up, were the main end points. To judge all incidents reported up to 24 h, a separate clinical event committee was constituted. The initial event for patients who experienced several occurrences was chosen for statistical modeling.

#### Covariates

Life style characteristics were discovered to be the independent risk factors for plaque vulnerability in earlier investigations on the relationship between Type D personality and coronary plaque vulnerability (5). The following factors were consequently taken into account while conducting the analysis: age, sex, hypertension, diabetes mellitus, hyperlipidemia, body mass index (BMI), smoking status (never, former, and current; assessed by self-report), and adherence to antiplatelet medication.

### Statistical analyses

The mean and standard deviation for normally distributed variables and the median (interquartile range) for skewed variables are used to describe the study population’s characteristics. The Mann-Whitney *U* test was used to analyze skewed variables, while the students’ *t*-test was employed to analyze variables with regularly distributed distributions. The χ^2^ test was used to compare counts (percentages) of categorical data. In order to do statistical analysis, hs-CRP, IL-6, and TNF-α were transformed to log 10 due to the skewed distributions of inflammatory biomarkers.

The correlations between Type D personality, KTR, inflammatory markers, plaque vulnerability characteristics, and MACE were examined by the Pearson or Spearman rank coefficients appropriately. Multivariate regression models were performed to determine the effect of Type D personality, KTR, inflammation markers on plaque vulnerability and MACE. Age, sex, hypertension, diabetes mellitus, hyperlipidemia, BMI, smoking status, and antiplatelet therapy adherence were entered in the regression analysis as covariates. In order to verify the reliability of results, a *post hoc* power analysis was performed.

We used regression-based mediation analysis, as reported by Preacher and Hayes, to examine the mediating roles of KTR and inflammatory markers on the link between Type D and plaque vulnerability/MACE (15). For the test from the Serial-Multiple Mediation Model 6, Hayes advised using 10,000 bootstrap bias-corrected 95 percent confidence intervals (CI) for mediation analysis. If the 95 percent CI excludes 0, then an effect is deemed substantial (16). PROCESS macro was performed using an independent variable (Type D personality), mediating variables (inflammation markers and KTR), and a dependent variable (plaque vulnerability and MACE). The core hypothesis model we tested was on how Type D personality influences plaque vulnerability and MACE through inflammatory markers and KTR. Both the direct and total effect were evaluated in accordance with Mathieu and Taylor’s (17) standards. Age, sex, and CAD risk factors (hypertension, diabetes, hyperlipidemia, BMI, smoking status, and adherence to antiplatelet medication) were then taken into account as variables.

## Results

### Sample characteristics

In all, 177 patients (73 women and 104 men, with an average age of 55.79 ± 10.70 years participated in the study. Seventy of them (or 39.5%) have Type D personality (Table 1). Between these two groups, there were no discernible variations in the demographic or clinical traits.

**TABLE 1:** Patients characteristics in Type D and non-Type D patients.

Variable	Total sample (*n* = 177)	Type D (*n* = 70)	Non-Type D (*n* = 107)	Test values	*P*-value
**Demographic and clinical characteristics**					
Age, M (SD), year	55.79 (10.70)	55.61 (11.14)	55.91 (10.45)	*t* = 0.85	0.86
Male, n (%)	104 (58.8)	42 (60.0)	62 (57.9)	χ^2^ = 0.07	0.78
Hyperlipidemia, n (%)	65 (36.7)	31 (44.3)	34 (31.8)	χ^2^ = 2.86	0.09
Diabetes, n (%)	33 (18.6)	17 (24.3)	16 (15.0)	χ^2^ = 2.43	0.12
Hypertension, n (%)	65 (36.7)	28 (40.0)	37 (34.6)	χ^2^ = 0.53	0.46
Smoking, n (%)	106 (59.9)	45 (64.3)	61 (57.0)	χ^2^ = 0.93	0.33
BMI, M (SD), kg/m^2^	25.14 (3.97)	25.76 (3.80)	24.72 (4.04)	*t* = 1.718	0.08
Antiplatelet therapy adherence, n (%)	155 (87.5)	60 (85.7)	95 (88.8)	χ^2^ = 0.36	0.64
**Laboratory markers**					
TNF-α, M (IQR), ng/L	9.53 (3.46)	10.18 (7.24)	9.32 (2.55)	*z* = –2.72	0.007
IL-6, M (IQR), pg/mL	18.21 (10.91)	19.57 (16.82)	17.91 (9.49)	*z* = –1.96	0.049
hs-CRP, M (IQR), mg/L	4.74 (8.49)	5.66 (10.29)	4.58 (6.61)	*z* = –1.32	0.18
Inflammation sumscores	–0.40 (2.63)	0.45 (2.72)	–0.76 (2.40)	*z* = –3.08	0.002
Tryptophan, M (IQR), μmol/L	10348.43 (4006.85)	10245.38 (3525.24)	10650.76 (3952.81)	*z* = –1.54	0.12
Kynurenine, M (IQR), μmol/L	303.44 (215.78)	353.86 (243.03)	278.78 (226.68)	*z* = –2.63	0.008
KTR, M (IQR), (×10^3^)	2.93 (1.17)	3.72 (2.72)	0.45 (2.72)	*z* = –2.82	0.005

TNF-α, tumor necrosis factor-α; IL-6, interleukin-6; hs-CRP, hs-C reactive protein; KTR, kynurenine-to-tryptophan ratio; M (IQR), median (interquartile range); M (SD), mean (standard deviation).

### The association of Type D personality with inflammation markers and kynurenine metabolite

In comparison to non-Type D people, Type D people had greater levels of TNF-α (*p* = 0.007), IL-6 (*p* = 0.049), inflammatory sumscores (*p* = 0.002), kynurenine (*p* = 0.008), and KTR (*p* = 0.005).

### The association of Type D personality with vulnerable plaque and major adverse cardiac events

Regarding the OCT characteristics of the two groups, plaque vulnerability characteristics of the Type D personality group, including the proportion of TCFA (77.1 vs. 50.5%, *p* < 0.001), macrophages (78.6 vs. 57.9%, *p* = 0.005), and rupture (68.6 vs. 39.3%, *p* < 0.001), were obviously higher than in the non-Type D personality group (Table 2). Furthermore, a total of 41 patients had MACEs during the 2-year follow-up period. The results showed that the percentages of MACE in the Type D were substantially higher than non-Type D groups (38.6 vs. 13.1%, *p* < 0.001).

**TABLE 2:** OCT analysis and MACE in Type D and non–Type D patients.

Variable	Total sample (*n* = 177)	Type D (*n* = 70)	Non-Type D (*n* = 107)	Test values	*P*-value
**OCT characteristics**					
Lipid plaque, n (%)	127 (71.8)	56 (80.0)	71 (66.4)	χ^2^ = 3.89	0.049
TCFA, n (%)	108 (61.0)	54 (77.1)	54 (50.5)	χ^2^ = 12.69	<0.001
Macrophage, n (%)	108 (66.1)	54 (77.1)	54 (50.5)	χ^2^ = 12.69	<0.001
Rupture, n (%)	90 (50.8)	48 (68.6)	42 (39.3)	χ^2^ = 14.55	<0.001
Thrombus, n (%)	150 (84.7)	57 (81.4)	93 (86.9)	χ^2^ = 0.98	0.32
MACE, n (%)	41 (23.2)	27 (38.6)	14 (13.1)	χ^2^ = 15.45	<0.001

OCT, Optical Coherence Tomography; TCFA, thin cap fibroatheroma; MACE, major adverse cardiac events.

The OCT indicators were used as dependent variables in the multivariate logistic regression model (Table 3). Following the adjustment for other pertinent covariates, it was discovered that Type D personality was an independent risk factor for TCFA (odds ratio [OR] = 2.46, 95% confidence interval [CI] = 1.07–5.64, *p* = 0.033), macrophage (*OR* = 2.41, 95% *CI* = 1.12–5.19, *p* = 0.025), and rupture (*OR* = 2.39, 95% *CI* = 1.11–5.16, *p* = 0.026).

**TABLE 3:** Multivariate regression model for coronary plaque vulnerability and MACE.

Variable	OR	95% CI	*P*-value
**Model 1: Dependent variable = TCFA**			
Type D personality	2.46	1.07–5.64	0.033
Inflammation sumscores	1.29	1.07–1.56	0.008
KTR	1.28	1.04–1.60	0.023
**Model 2: Dependent variable = Macrophage**			
Type D personality	2.41	1.12–5.19	0.025
Inflammation sumscores	1.27	1.02–1.58	0.036
KTR	1.31	1.07–1.61	0.008
**Model 3: Dependent variable = Rupture**			
Type D personality	2.39	1.11–5.16	0.026
Inflammation sumscores	1.26	1.01–1.56	0.037
KTR	1.35	1.10–1.65	0.004
**Model 4: Dependent variable = MACE**			
Type D personality	2.42	1.04–5.60	0.038
Inflammation sumscores	1.30	1.07–1.58	0.007
KTR	1.26	1.02–1.58	0.035

KTR, kynurenine-to-tryptophan ratio; TCFA, thin cap fibroatheroma; MACE, major adverse cardiac events; OR, odds ratio; CI, confidence interval. The models were adjusted for traditional cardiovascular risk factors including age, sex, hypertension, diabetes mellitus, hyperlipidemia, BMI, smoking status, and antiplatelet therapy adherence.

Type D personality was then added into the multivariate logistic regression analyses. The results indicated that after adjusting for age, sex and other traditional cardiovascular risk factors, Type D personality (*OR* = 2.42; 95% *CI* = 1.04–5.60; *p* = 0.038) was independent predictor of 2-year MACEs (Table 3). Furthermore, the *post hoc* power analysis for multiple regression of TCFA, macrophage, rupture, and MACE is 95.6, 95.6, 97.6, and 97.2%, which indicated that the regression results are reliable.

### Inflammation markers, kynurenine metabolite, vulnerable plaque, and major adverse cardiac events

From the bivariate correlation analyses in Table 4, the results showed that TNF-α and inflammation sumscores were positively correlated with TCFA, macrophage, rupture and MACE. Furthermore, IL-6 was positively correlated with TCFA and macrophage.

**TABLE 4:** Correlations between Type D Personality, inflammation sumscores, KTR, and plaque vulnerability.

Variable	1	2	3	4	5	6	7	8	9	10	11	12
Type D personality	–	0.216**	0.144	0.144	0.294**	–0.003	0.244**	0.252**	0.267**	0.267**	0.287**	0.295**
TNF-α		–	0.335**	–0.034	0.634**	0.059	0.225**	0.205**	0.254**	0.234**	0.253**	0.333**
IL-6			–	0.297**	0.748**	–0.064	0.081	0.083	0.161*	0.215**	0.127	0.184*
hs-CRP				–	0.585**	–0.053	0.103	0.067	0.094	0.084	0.099	0.005
Inflammation sumscores					–	–0.048	0.218**	0.218**	0.284**	0.275**	0.250**	0.327**
Tryptophan						–	0.014	–0.170*	–0.051	–0.088	0.028	–0.008
Kynurenine							–	0.848**	0.276**	0.351**	0.210**	0.172*
KTR								–	0.275**	0.326**	0.201**	0.269**
TCFA									–	0.430**	0.651**	0.247**
Macrophage										–	0.373**	0.247**
Rupture											–	0.165*
MACE												–

TNF-α, tumor necrosis factor-α; hs-CRP, hs-C reactive protein; IL-6, interleukin-6; KTR, kynurenine-to-tryptophan ratio; TCFA, thin cap fibroatheroma; MACE, major adverse cardiac events. *p < 0.05; **p < 0.01.

For kynurenine metabolite, kynurenine, and KTR were positively correlated with TCFA, macrophage, rupture, and MACE.

According to the modified multivariate regression model, inflammation sumscores (*OR* = 1.29, 95% *CI* = 1.07–1.56) (*OR* = 1.27, 95% *CI* = 1.02–1.58) (*OR* = 1.26, 95% *CI* = 1.01–1.56) (*OR* = 1.30, 95% *CI* = 1.07–1.58) and KTR (*OR* = 1.28, 95% *CI* = 1.04–1.60) (*OR* = 1.31, 95% *CI* = 1.07–1.61) (*OR* = 1.35, 95% *CI* = 1.10–1.65) (*OR* = 1.26, 95% *CI* = 1.02–1.58) were independent predictors of TCFA, macrophage, rupture, and MACEs (Table 3).

### Mediation analyses of Type D personality on vulnerable plaque and major adverse cardiac events-the mediation effect of inflammation sumscores and kynurenine/tryptophan ratio

**FIGURE 2:**
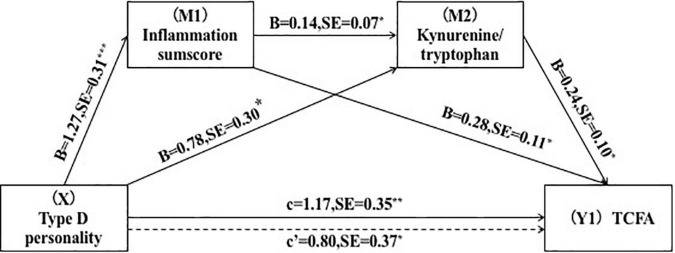
Serial-multiple mediation of inflammation and kynurenine/tryptophan ratio in the relationship between Type D personality and TCFA. *p < 0.05, **p < 0.01.

**FIGURE 3:**
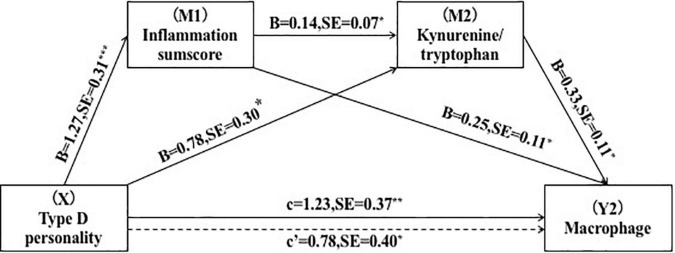
Serial-multiple mediation of inflammation and kynurenine/tryptophan ratio in the relationship between Type D personality and macrophage. *p < 0.05, **p < 0.01.

To ascertain the serial-multiple mediation of inflammation and the kynurenine metabolite in the link between Type D personality and plaque vulnerability, we conducted SPSS Macro mediating analyses. We included plaque vulnerability characteristics, including TCFA, macrophages, and rupture as dependent variables in the mediation models. The result demonstrated that inflammatory sumscores and KTR mediated the association between Type D and plaque vulnerability when all covariables in the tested model were taken into account (Y1 = TCFA, Y2 = macrophages) (Table 5). The path through single mediation of inflammation sumscores (Y1: point estimate = 0.37; 95% *CI* = 0.09–0.79) (Y2: point estimate = 0.33; 95% *CI* = 0.07–0.73), single mediation of KTR (Y1: point estimate = 0.05; 95% *CI* = 0.03–0.12) (Y2: point estimate = 0.05; 95% *CI* = 0.06–0.17), and both mediators (Y1: point estimate = 0.19; 95% *CI* = 0.03–0.50) (Y2: point estimate = 0.26; 95% *CI* = 0.05–0.63) were all found to be statistically significant. Furthermore, the direct effects of Type D personality on TCFA (point estimate = 0.81; 95% *CI* = 0.09–1.53) and macrophages (point estimate = 0.79; 95% *CI* = 0.05–1.51) was also found to be statistically significant (Figures 2, 3).

**TABLE 5:** The effects of Type D personality on plaque vulnerability and MACE mediated by inflammation and KTR.

Dependent variable	Direct and mediation effect paths	Product of coefficients		Bootstrapping 95% BC confidence interval (CI)	
		Point estimate	Boot SE	BootLL CI	BootUL CI
Y1 = TCFA	**Direct effect of X on Y1**	0.81	0.37	0.09	1.53
	**Indirect effect of X on Y1**	0.61	0.21	0.27	1.11
	Indirect effect 1: X → M1 → Y1	0.37	0.18	0.09	0.79
	Indirect effect 2: X → M1 → M2 → Y1	0.19	0.12	0.03	0.50
	Indirect effect 3: X → M2 → Y1	0.05	0.03	0.03	0.12
Y2 = Macrophage	**Direct effect of X on Y2**	0.79	0.37	0.05	1.51
	**Indirect effect of X on Y2**	0.65	0.23	0.30	1.18
	Indirect effect 1: X → M1 → Y2	0.33	0.17	0.07	0.73
	Indirect effect 2: X → M1 → M2 → Y2	0.26	0.15	0.05	0.63
	Indirect effect 3: X → M2 → Y2	0.05	0.04	0.06	0.17
Y3 = MACE	**Direct effect of X on Y3**	0.88	0.42	0.08	1.70
	**Indirect effect of X on Y3**	0.56	0.21	0.24	1.07
	Indirect effect 1: X → M1 → Y3	0.33	0.17	0.07	0.73
	Indirect effect 2: X → M1 → M2 → Y3	0.19	0.11	0.02	0.46
	Indirect effect 3: X → M2 → Y3	0.04	0.03	0.01	0.13

M1, inflammation sumscores; M2, kynurenine-to-tryptophan ratio. The direct effect shows the direct effects of Type D personality on plaque vulnerability and MACE when the mediators (inflammation and KTR) is included in the model. The indirect effect shows the indirect effects of Type D personality on plaque vulnerability and MACE via the mediators. The indirect (mediation) effect is significant if the bootstrapped confidence intervals do not include 0. LLCI, Limited liability confidence interval; ULCI, Upper liability confidence interval.

**FIGURE 4:**
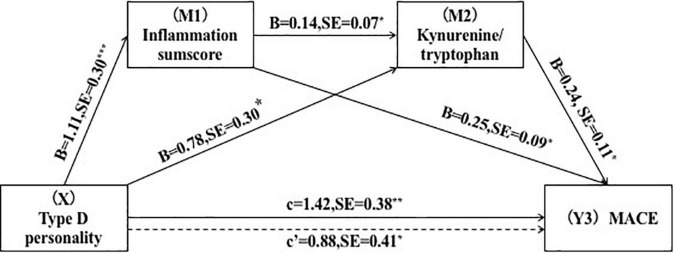
Serial-multiple mediation of inflammation and kynurenine/tryptophan ratio in the relationship between Type D personality and MACE. *p < 0.05, **p < 0.01.

Furthermore, we conducted mediating studies to see if inflammation and the kynurenine metabolite were possible mediators of the relationship between Type D personality and MACE. According to the findings, the single mediation of inflammation sumscores (point estimate = 0.33; 95% *CI* = 0.07–0.73), the single mediation of KTR (point estimate = 0.04; 95% *CI* = 0.01–0.13), and the combined mediation (point estimate = 0.20; 95% *CI* = 0.10–0.58) was significant. The direct effects of Type D personality on MACE (point estimate = 0.88, 95% *CI* = 0.08–1.70) were as striking after adjusting for variables (Figure 4).

## Discussion

In this study, we found that the Type D personality group exhibited elevated levels of TNF-α, IL-6, inflammation sumscores, kynurenine, and KTR. Furthermore, mediation analyses revealed that elevated inflammation sumscores and KTR levels mediated the effect of Type D personality on coronary vulnerable plaque and MACE. Our findings provide a theoretical framework for comprehending how Type D personality influences the onset and prognosis of CAD.

First, our results suggest that Type D personality has a direct effect on vulnerable plaque (TCFA and macrophage) and 2-year follow-up MACE. The local inflammatory milieu and plaque vulnerability are controlled by the macrophage by secreting inflammatory chemicals, cytokines, and growth factors. TCFA is a significant predictor of atherosclerotic plaque vulnerability (18). Our previous OCT studies confirmed that Type D personality is an independent risk factor for coronary plaque (4, 19). Moreover, other OCT studies confirmed that the presence of plaques *in vivo* enhanced MACE risk in CAD patients (20). Our results further demonstrated that the increased occurrence of MACE in Type D patients can be partially attributed to lower plaque stability. However, the mechanism underlying Type D personality influencing plaque vulnerability and poor prognosis has not been established.

Using the serial-multiple mediation model’s findings, we found that Type D personality can influence vulnerable plaque and follow-up MACE through inflammatory biomarkers. We found that the Type D group exhibited significantly elevated levels of TNF-α, IL-6, and inflammation sumscores. This observation was in tandem with a previous research, it claimed that inflammatory activation was elevated in people with Type D personality (21, 22). Denollet et al. confirmed that the immune system can be significantly activated by Type D personality, which is manifested by elevated plasma biomarkers of inflammation (hs-CRP, IL-6, IL-8, TNF-α, etc.) (13). Furthermore, inflammation plays a key role in CAD initiation and progression and in enhancing coronary plaque vulnerability. A recent meta-analysis showed that the TNF-α and IL-1 family were involved in plaque formation and, consequently, in the incidences of myocardial infarction. Moreover, IL-6 has a significant prognostic value in CAD patients (23). Therefore, our findings confirmed that the Type D personality can affect the dynamic progression of CAD mediated by pro-inflammatory factors.

In addition, path mediation analysis revealed that the activation of tryptophan metabolism along the kynurenine pathway partly mediated the effect of Type D on plaque vulnerability and MACE. Targeted metabolomics showed that kynurenine and KTR levels were elevated in Type D personality patients. However, a previous non-targeted metabolomics study found that Type D personality had significantly lower levels of kynurenine (24). This inconsistency may be attributed to different metabolomics measurement, for targeted metabolites can achieve absolute quantification of metabolites. Moreover, our analysis is substantially more comprehensive as it includes the metabolism of tryptophan into the kynurenine pathway, which can reflect the activity of IDO enzyme (12). Studies have documented that elevated IDO activity is positively correlated with endothelial dysfunction and carotid artery intima-media thickness (25). A large cohort study confirmed that KTR levels are relatively stable over time, and was identified as a particularly strong predictor of acute myocardial infarction, all-cause and cardiac mortality in CAD patients (9). Therefore, the kynurenine tryptophan metabolism pathway can be used to clarify the underlying mechanisms between Type D personality, plaque vulnerability and MACE.

Furthermore, Type D personality was shown to activate kynurenine metabolism through inflammatory reactions, which led to the formation of vulnerable plaque and the occurrence of MACE. High inflammation may indeed, in fact, speed up the conversion of tryptophan to kynurenine (26). Interferon-, a pro-inflammatory cytokine, and lipopolysaccharide, to a lesser extent, both highly activate IDO, an enzyme that catalyzes tryptophan metabolism along the kynurenine route (8). This study proved that Type D personality had higher inflammation levels, which speed up kynurenine metabolism. High KTR levels can therefore predict the development of susceptible plaque and MACE. Therefore, inhibition of inflammatory levels in Type D individuals can help maintain kynurenine metabolic balance, improve plaque stability and ultimately reduce MACE.

## Limitations

Several limitations in our study should be noted. First, this is a single center using a small sample size. More large-scale studies from multiple centers are needed for improved statistical power. Second, glucocorticoid levels were not measured in this study, therefore, the TDO enzyme in the metabolism of tryptophan to kynurenine cannot be reflected. Third, depression, anxiety, and stress-related symptoms was not measured in this study, which should be measured and adjusted in the further research in order to confirm our conclusion. Finally, results from our study among CAD population may not be generalizable to other populations.

## Conclusion

To the best of our knowledge, this is the first study to use a serial-multiple mediation model to assess the underlying mechanisms of Type D personality on cardiovascular outcomes. We found that pro-inflammatory biomarkers and high KTR can mediate the effect of Type D personality on coronary vulnerable plaque and MACE. These results are especially significant given that indications for poor cardiovascular prognosis in Type D group can involve inflammation and the kynurenine pathway.

## Data availability statement

The original contributions presented in this study are included in the article/supplementary material, further inquiries can be directed to the corresponding authors.

## Ethics statement

The studies involving human participants were reviewed and approved by the Ethics Committee of the Second Affiliated Hospital of Harbin Medical University. The patients/participants provided their written informed consent to participate in this study.

## Author contributions

YW: methodology and writing—original draft. XG and GL: investigation and methodology. SY, LL, and XS: formal analysis. ZZ and BY: resources and data curation. PL: conceptualization and funding acquisition. YY: project administration and supervision. All gave final approval and agree to be accountable for all aspects of work ensuring integrity and accuracy.

## Footnotes

1
http://www.magigene.com

2
http://www.xjqxjl.com
